# Relationship between participants’ level of education and engagement in their completion of the Understanding Dementia Massive Open Online Course

**DOI:** 10.1186/s12909-015-0344-z

**Published:** 2015-03-26

**Authors:** Lynette R Goldberg, Erica Bell, Carolyn King, Ciaran O’Mara, Fran McInerney, Andrew Robinson, James Vickers

**Affiliations:** 1 Wicking Dementia Research and Education Centre, School of Medicine/Faculty of Health, University of Tasmania, Private Bag 143, 7001 Hobart, Tasmania Australia; 2 School of Medicine, University of Tasmania, Private Bag 34, 7001 Hobart, Tasmania Australia

**Keywords:** Dementia, Online learning, MOOC, Level of education, Engagement

## Abstract

**Background:**

The completion rates for Massive Open Online Courses (MOOCs) generally are low (5-10%) and have been reported to favour participants with higher (typically tertiary-level) education. Despite these factors, the flexible learning offered by a MOOC has the potential to provide an accessible educational environment for a broad spectrum of participants. In this regard, the Wicking Dementia Research and Education Centre has developed a MOOC on dementia that is evidence-based and intended to address this emerging major global public health issue by providing educational resources to a broad range of caregivers, people with dementia, and health care professionals.

**Methods:**

The Understanding Dementia MOOC was designed specifically to appeal to, and support, adult learners with a limited educational background. The nine-week course was presented in three units. Participants passed a quiz at the end of each unit to continue through the course. A series of discussion boards facilitated peer-to-peer interactions. A separate “Ask an Expert” discussion board also was established for each unit where participants posted questions and faculty with expertise in the area responded.

**Results:**

Almost 10,000 people from 65 countries registered; 4,409 registrants engaged in the discussion boards, and 3,624 (38%) completed the course. Participants’ level of education ranged from postgraduate study to a primary (elementary) school education. Participants without a university education (vocational certificate and below) were as likely as those with a university education to complete the course (χ^2^ = 2.35, *df* = 6, *p* = 0.88) and to engage in the online discussions (*F*[6, 3799] = 0.85, *p* = 0.54). Further, participants who completed the MOOC engaged in significantly more discussion board posts than participants who did not complete the course (*t* = 39.60, *df* = 4407, *p* <0.001).

**Conclusions:**

The high completion rate and level of engagement of participants across a broad spectrum of levels of education suggest that MOOCs can be successfully developed and delivered to students from diverse educational backgrounds. The high participation rate also highlights the combination of MOOC design as well as the scale of unmet need for quality dementia education.

## Background

Massive Open Online Courses (MOOCs) provide free Internet-based learning opportunities and came to prominence in 2012 [1]. These courses have been developed independently by university-based academics or as part of contractual agreements between higher education institutions and third party online platforms, and have been successful in attracting tens of thousands of students [2]. Proponents suggest that MOOCs can be considered part of the Open Educational Resources (OER) movement as they provide free educational offerings to anyone at any location [3]. With no formal requirements for entry, MOOCs aim to promote inclusiveness, equity in educational opportunities, and valuable autonomous and peer-to-peer learning opportunities [4]. They are viewed as complementing traditional university education rather than replacing it, although a MOOC-based university is planned for Rwanda, with the associate degrees from this university being certified by a university in the United States [5,6]. Critics of MOOCs have expressed concern that the massive number of enrolled students makes it difficult for instructors to implement effective teaching and learning strategies, provide consistent feedback and guidance, and obtain meaningful evaluative data. In addition, they feel that the emphasis on technology and logistics may broadly overwhelm teaching and learning. Some universities that offered MOOCs now have returned to smaller online courses with a closed application process [7]. However, even critics agree that: “…a few efficient MOOCs may go quietly about the business of offering distance education…and provide a useful bridge between casual informal learning and formal study” ([8], p. 130).

Siemens [9] and Downes [10], the developers of the original MOOC concept, proposed a theory of connectivism whereby students learn to become astute and autonomous consumers in finding information rapidly through a technological and social network of multiple connections and experiences. However, these investigators recognized that students with a limited educational background might be at a disadvantage in online learning environments, particularly in managing the requisite technology. Advancing this theory, Kop, Fournier, and Mak [11] emphasized the idea of “emergent learning” as learning in which participants and the technology-based system co-evolve. Although a large electronic forum can potentially act as a barrier to students’ connecting or identifying with the learning medium, participation in MOOCs through authentic and meaningful learning experiences has been shown to facilitate autonomy, critical reflection, inclusivity, dialogue, self-development, and a sense of community – all important elements of cognitive and social constructivism in students’ continued situated learning [9,11-13].

One intent of MOOCs has been to provide pre-requisite education to enable students to begin and successfully complete more traditional higher education programs [14]. However, despite their intended inclusivity, data show that MOOCs have largely been taken up by traditional university students (including students with limited resources who may not be able to attend an elite school), and graduates, rather than non-traditional students from limited educational backgrounds [15]. From a survey of almost 35,000 MOOC participants, Emanuel et al. [16] reported that more than 80% of these students had a two- or four-year university degree. This also held true for students from less well-developed countries. Thus, to date, MOOCs appear to favour those with more education rather than making online education more accessible and equitable.

Regardless of content or university experience, the percentage of students completing MOOCs has been low, frequently between 5 and 10%, even for students with university degrees [17-21]. As requirements for completion vary across courses, completion rates have been calculated as the percentage of students (out of the total course enrolment) who received a certificate for the course [20]. Low completion rates are associated with longer course length, with courses ranging from 5–25 weeks, and may also reflect the degree to which students are actively engaged in the teaching and learning process, self-directed in their learning, able to access the needed technology, and supported by faculty [20,22].

In this regard, Seaton and his colleagues [23] tracked the activity of 154,000 MOOC registrants during a spring semester in 2012. Of the 108,000 participants who accessed the course, only 6% completed it. Non-completers (94%) invested less time in the early weeks of the course, attempted fewer assessments and spent less time on them than completers (6%). Completers spent a notable amount of time on the socially-oriented discussion forums and their discussion activity increased over the semester. Sixty percent of the total time tracked for the course was invested by the 6% of students who completed it.

In an examination of the teaching and learning practices in 24 university-level MOOCs, Toven-Lindsey and colleagues [24] found all courses used the one-way instructional approach common to face-to-face learning, where the instructor (the expert) transfers information to the learner (the novice). In some of the courses with specified start and end dates, students moved through the course at the same time and these courses included collaborative activities to facilitate student engagement. Across MOOCs, the online discussion boards again proved to be a valuable tool to encourage peer interactions and threaded conversations, often in response to open-ended posts from the instructor [24,25].

Universities are increasingly being encouraged to achieve greater participation from students who come from socio-economically and educationally disadvantaged backgrounds. Many of these students are mature age adults who are employed in lower level positions due to their limited education and lower-level qualifications [26]. These adult learners require flexible learning opportunities which are, in theory, accessible through a MOOC. Studies show that these adult learners tend to be internally motivated, self-directed, and goal-oriented [27-29]. The multiple roles they frequently manage - including working full- or part-time, being a parent, spouse or partner, and possibly a caregiver - can enrich, as well as challenge, the learning situation [30].

The recruitment of students with vocational qualifications is of particular importance in increasing the education of those who care for people with dementia. This age-related condition is an emerging major global public health issue [31-33] and caregivers frequently are not required to have experience or formal qualifications to work in this area [32,34]. As a result, their knowledge of dementia and approaches to care is limited [35]. In the context of the world-wide increase in the number of adults over the age of 65 years, there is a need for all members of society to understand issues in ageing, and how dementia is not part of typical ageing but rather involves progressive and degenerative pathology in the brain. Further, it is important that people understand that there are current and available best practice approaches to care based on the staging of dementing illnesses [35]. To address these issues, the Wicking Dementia Research and Education Centre (WDREC) at the University of Tasmania (Australia) developed a MOOC focused on “Understanding Dementia” [36-38]. The course is open to all, but designed specifically to also appeal to, and support, adult learners with limited educational backgrounds. The development of this MOOC came from extensive research and market investigation by the WDREC, including consultation with dementia care consumers and care organizations. The MOOC also serves as a pathway into the newly-established and fully online diploma, associate degree and full-degree Bachelor of Dementia Care programs.

### The Understanding Dementia MOOC

Almost 10,000 people registered for the first offering of the course in 2013 and 38% successfully completed it. This completion rate is notably better than the completion rates reported for other MOOCs [20,21]. The nine-week course was divided into three-week units and conducted between July and October. There was a staggered release of each unit with a one-week break between each release. All units remained open through November to allow participants time to compete the course. The first unit, “The Brain,” considered basic concepts in nervous system anatomy and function, the pathology underlying dementia, and current and future research into the varying presentations of dementia. The second unit, “The Disease,” explored the differences between typical ageing and dementia, risk factors for dementia, symptoms, diagnosis, and medical management. The third unit, “The Person,” considered the insidious onset of dementia, living with dementia, the progression and stages of the condition, associated behavioral changes, non-pharmacological management, and issues in palliation. Within each unit, participants watched and listened to a series of short videos of experts discussing dementia, complemented by the presentation of authentic cases and interactive learning activities.

As each unit was posted, participants could move through the activities at their own pace. They were encouraged to post their questions and comments as they completed each activity. A series of discussion boards facilitated peer-to-peer interactions. A separate “Ask an Expert” discussion board was established for each unit, where participants posted questions and faculty with expertise in the area responded. Posting to either type of discussion board was optional and participants could identify themselves or remain anonymous. At the end of each unit, participants completed a 20-item multiple-choice quiz. Participants could re-take this quiz as many times as was necessary to achieve the passing grade of 70% and progress to the next unit. After successfully completing the three units, participants were awarded a Certificate of Completion.

Based on reports related to accessibility for other MOOCS, the current study focuses on whether completion of the MOOC related to participants’ level of education and relative level of engagement through discussion posts. The following research questions were posed: (1) Is there a difference between participants who did and did not complete the MOOC with regard to their level of education? (2) Does level of education affect the number of discussion posts participants make? And (3) is there a difference between the number of discussion posts made by participants who completed and did not complete the MOOC?

## Methods

### Procedure

To promote the course and develop an online presence, advertisements were posted on Facebook, web forums and blogs focused on aged care, and MOOC sites. Emails about the course also were sent to administrators of Residential Aged Care Facilities (RACFs; elsewhere known as nursing homes or Continuing Care Retirement Communities) across Australia and internationally. Participants accessed the course via a self-registration module, using existing Desire2Learn software, made available through the University.

### Participants

The free online course was open to any interested person. When participants enrolled in the course, they were asked to complete a short biographical questionnaire that included questions about their geographical location, level of education and experience with people with dementia. Participants were welcomed to the MOOC on a Social Space and were encouraged to introduce themselves and begin to engage with one another.

### Data analysis

Ethics approval for the study was obtained from the Tasmanian Social Science Human Research Ethics Committee (ref: H0013173). At the end of the course, data were grouped using MySQL software. Descriptive data were used to identify participants’ level of education, MOOC completion, and the number of discussion board posts. There were seven levels of education (postgraduate degree, Bachelor degree, associate degree, vocational certificate, upper secondary school, lower secondary school, and primary/elementary school) plus “unknown.” The known levels of education were categorized into two groups: university degree and no university degree, for statistical analysis using chi-square. Participants’ discussion posts were collated into a text file. Posts made by MOOC faculty and support staff were excluded. Analysis of variance (ANOVA) and *t*-test procedures were used to investigate the number of discussion posts according to participants’ level of education and whether or not they had completed the MOOC.

## Results

The 9,538 registrants for the first iteration of the Understanding Dementia MOOC represented 65 known countries: 4,254 participants came from countries in the Organisation for Economic Co-operation and Development (OECD) forum and 134 came from non-OECD countries. The remaining participants did not specify their country of origin. Of the 9,538 registrants, 3,624 (38%) completed the course. These “completers” were predominantly female (female = 3,183; male = 303; unknown = 138) and 1,398 (26%) of these completers were ≥ 50 years of age. The known levels of education of the completers ranged from postgraduate study (n = 529) to a primary school education (n = 17): 1,037 had completed an undergraduate degree and 800 had received a vocational certificate. The number and education level of those who did and did not complete the MOOC are summarized in Table 1. Chi-square analysis documented no significant difference in MOOC completion between participants who had a university degree and those who did not (χ^2^ = 2.35, *df* = 6, *p* = 0.88). Thus, participants without a university education (vocational certificate and below) were as likely as those with a university education to complete the course.

**Table 1:** **Completion rates of participants in the Understanding Dementia MOOC by level of education**

Level of education	# who did not complete the MOOC	# who completed the MOOC
University degree		
Postgraduate degree	354	529
Bachelor degree	717	1,037
Associate degree	95	168
Subtotal	1,166	1,734
No university degree		
Vocational certificate	544	800
Upper secondary school	267	383
Lower secondary school	100	146
Primary school	11	17
Subtotal	921	1,345
Unknown	3,826	544
Total	5,914	3,624

Of the cohort of 9,538 MOOC registrants, 4,409 engaged in the online discussion forums. Of these, 2,896 completed the MOOC and 1,513 did not. These 4,409 participants made 45,955 posts. These participants and their posts are presented by level of education in Table 2. Participants who posted on discussion boards represented all levels of education: 2,143 had completed a university degree; 1,663 did not have a university degree. Participants with a university degree made 23,128 posts; those without a degree made 18,453 posts. Results of the ANOVA confirmed there were no significant differences between the number of discussion posts made by participants with a university degree compared to the number of posts made by those without a university education (*F*[6, 3799] = 0.85, *p* = 0.54). Thus, discussants with lower levels of education appeared as likely as those with university experience to engage in the online discussions. An analysis of the number of discussion posts made by completers compared to the number of posts made by non-completers confirmed a significant difference between these two groups (*t* = 39.60, *df* = 4407, *p* <0.001). As is evident in Table 2, completers made more posts than non-completers across all levels of education.

**Table 2:** **MOOC discussion participants and posts by level of education**

Level of education	# of discussion participants		# of posts		Mean # posts ± standard deviation made by completers
	C	NC	C	NC	
University degree					
Postgraduate degree	447	217	6,563	733	10.99 ± 0.79
Bachelor degree	860	411	11,899	1,455	10.51 ± 0.55
Associate degree	147	61	2,214	264	11.91 ± 1.39
Subtotal	2,143		23,128		
No university degree					
Vocational certificate	681	337	10,169	1,139	11.11 ± 0.63
Upper secondary school	313	144	4,597	451	11.05 ± 0.96
Lower secondary school	111	56	1,678	214	11.33 ± 1.55
Primary school	16	5	191	14	9.76 ± 4.05
Subtotal	1,663		18,453		
Unknown	321	282	3,620	754	
Total	4,409		45,955		

Note. C = completer; NC = non-completer of the MOOC.

## Discussion

The expectation from previous studies that participants with a university education would be more likely to complete the Understanding Dementia MOOC and engage in discussion posts compared to participants without a university education was not confirmed. Participants with education levels that ranged from primary (elementary) school to vocational training were as likely to complete this MOOC as participants with associate, undergraduate, and postgraduate university degrees. This is an important and previously un-reported finding. It supports the intent of MOOC developers to be inclusive and offer learning opportunities to students from diverse educational backgrounds to equip them for further university study [3,4]. It demonstrates the value of scaffolding the delivery of content for student-centred and student-student learning [37], and confirms that students do not have to have a university degree to negotiate required technology and become successful online learners [11]. Of equal importance, it verifies that a carefully developed MOOC can provide an authentic and needed learning experience for people who are caring for adults with dementia, particularly when these caregivers have limited educational experience [8,35,37,38].

Some investigators have argued that the size of the electronic online learning MOOC forum can be intimidating to potential students and inhibit their involvement [7-9]. Others have shown that a lengthy course and a static online one-way instructional approach are additional inhibiting factors [20,22,24,25]. The results of the current study demonstrate how an open online interactive medium can facilitate student engagement in the absence of a traditional learning space. The Understanding Dementia MOOC was nine weeks in length. Its three units were deliberately designed to present authentic and interactive learning opportunities in a social environment that involved multiple collaborative activities and faculty-supported discussion boards [24,25]. A further important finding from the study was the value of these active and supported discussion boards in engaging students [23,24,37]. Participants who completed the MOOC engaged in the discussion boards significantly more often than those who did not complete the course. Thus, the effectiveness of the Understanding Dementia MOOC appeared to reflect both the creativity and relevance of the scaffolded content and the variety of welcoming social learning environments, without space, time, and judgement constraints, particularly for students from diverse educational backgrounds [24,25,37].

Although the MOOC was comparatively short compared to the 5–25 week range reported in other studies [20,22], it required the continuous engagement of numerous Wicking Centre staff and associates with content expertise to support the learning and related explorations of participants. Such resource commitments necessitate a university-supported and sustainable business model that recognizes the possibility of achieving globally-significant educational outcomes [37]. While a sizable number of registrants did not complete the course, the percentage of participants who did was notably higher than the percentage completion rate reported for other MOOCs [17-21]. Further, as measured by the number of their discussion posts and across all levels of university and non-university education, participants who completed the MOOC were significantly more engaged in the learning opportunities on offer than non-completers. Thus, the format of the Understanding Dementia MOOC appeared to invite students from a variety of educational backgrounds and to facilitate, rather than impede, their participation and sustained engagement in learning. It is also likely that many participants had substantial professional (i.e., unmet development needs) and/or personal (family member affected by dementia) relationships with the field of dementia, which sustained their engagement [35].

These positive findings make a valuable contribution to the MOOC literature. However, work must continue to investigate why a majority of registrants did not complete the MOOC or participate actively in discussion board posts. It could be that the electronic format was a barrier for these registrants and they were not ready to learn in this way [11,12]. Alternatively, they may have begun the course with enthusiasm but then prioritized other activities. Some achieved 100% on the first quiz but did not attempt the second and third. Perhaps these participants felt insufficiently challenged by the level of knowledge on offer? Some achieved 65% on the third and final quiz but did not re-take the quiz to achieve the required 70% and so did not officially complete the course. Possibly these participants were satisfied with what they had learned and felt they did not need to continue, and/or require the certificate of completion. As difficult as it might be, such questions lend themselves to implementing post-MOOC completion/withdrawal evaluation surveys to document participant experiences, satisfaction, and feedback. Other important initiatives focus on delving into existing MOOC data to identify effective engagement strategies [39,40] including, for example, the value of using time-stamped logs of student behavior to gain insight into how, and on what, students spend their time when completing a MOOC [23].

In the current study, engagement was measured through the number of discussion board posts made by participants. While this is a viable measure, a possible deterrent to making, or continuing to make, discussion board posts could be a type of ceiling effect experienced by participants, in that what they might want to contribute has already been said. This introduces the possible influence of participant personality. In face-to-face conversations, some people repeat points already made, some begin a new topic of conversation, and some stay silent until they feel a need to comment. Parallel to this, in online discussions, participants can read and learn from posts but not make them, i.e., remain silent until an interesting issue prompts them to participate more actively. These participants are termed “lurkers” [11,41]. The measurement of lurkers is valuable as it differentiates participants who have dropped out of the course from those who are present, perhaps observing and listening, but not posting on discussion boards. This measurement also can reflect the theoretical underpinning of a MOOC – whether it is more connectivist (networking and learning) or cognitive-behaviorist (task-focused) in nature [9,10,41]. Future course evaluations might profit from an exploration of such types of engagement.

The Understanding Dementia MOOC was open to anyone interested in the topic of dementia. It was designed specifically to support adult learners with limited educational backgrounds with the expectation that many of these adult learners would be providing care to persons with dementia. It sought to increase participants’ awareness and understanding of dementia, challenge incorrect or stereotypical thinking, and encourage and sustain supportive networking. In this regard it was intended to be a comfortable place for both learning and lurking. For participants wishing to continue on to university study, completion of the MOOC was an entry point into a “bridging” unit of the Bachelor of Dementia Care program, a fully online degree also offered through the WDREC. In this way, for many participants, the Understanding Dementia MOOC formed the valuable pathway between informal and formal learning espoused by Baggaley [8].

## Conclusions

A 9-week Understanding Dementia MOOC was developed specifically to appeal to and support adult learners from a variety of educational backgrounds. Thirty-eight percent (3,624) of registrants completed the course. Participants’ level of education ranged from primary (elementary) school to postgraduate study. Participants without a university education (vocational certificate and below) were as likely as those with a university education to complete the course and to engage in the online discussions. Further, participants who completed the MOOC engaged in significantly more discussion board posts than participants who did not complete the course. The high completion rate and level of engagement of participants with lower levels of education suggest that MOOCs can appeal to students from diverse educational backgrounds and demonstrate that students do not have to have a university degree to be successful online learners.

## References

[1] Pappano L. The year of the MOOC. New York Times November 11, 2012. http://www.nytimes.com/2012/11/04/education/edlife/massive-open-online-courses-are-multiplying-at-a-rapid-pace.html?_r=0

[2] UK Universities (2013). Massive open online courses - higher education's digital moment?.

[3] Matkin GW. The opening of higher education. Change May/June, 2012. http://www.changemag.org/Archives/Back%20Issues/2012/May-June%202012/Higher%20Ed.full.html

[4] Gaebel M (2013). EUA occasional papers: MOOCs – Massive Open Online Courses.

[5] Allen EI, Seaman J (2013). Changing course: Ten years of tracking online education in the United States.

[6] Lucas H (2014). Disrupting and transforming the university. Comm ACM.

[7] Baggaley J (2013). MOOC rampant. Dist Educ.

[8] Baggaley J (2014). Reflection: MOOC postscript. Dist Educ.

[9] Siemens G. Connectivism: learning as a network-creation. ELearnspace 2005. http://www.elearnspace.org/blog/2005/08/11/connectivism-learning-as-network-creation/

[10] Downes S, Hug T (2007). An introduction to connective knowledge. Media, knowledge & education – Exploring new spaces, relations and dynamics in digital media ecologies: Proceedings of the International Conference.

[11] Kop R, Fournier H, Mak JSF (2011). A pedagogy of abundance or a pedagogy to support human beings? Participant support on massive open online courses. Int Rev Res Open Dist Learn.

[12] Deimann M, Farrow R (2013). Rethinking OER and their use: open education as bildung. Int Rev Res Open Dist Learn.

[13] Baxter JA, Haycock J (2014). Roles and student identities in online large course forums: implications for practice. Int Rev Res Open Dist Learning.

[14] Daza V, Makriyannis N, Rovira Riera C (2013). MOOC attack: closing the gap between pre-university and university mathematics. Open Learn.

[15] Christensen G, Alcorn B, Emanuel EJ. MOOCs won’t replace business schools – they’ll diversify them. Harvard Business Review Blog Network June 3, 2014. http://blogs.hbr.org/2014/06/moocs-wont-replace-business-schools-theyll-diversify-them

[16] Emanuel EJ (2013). MOOCs taken by educated few. Nature.

[17] Parkinson D (2014). Implications of a new form of online education. Nurs Times.

[18] McGettigan A. Will “Moocs” be the scourge or saviour of higher education? In: The Guardian, London, Scott Trust Publishers; 2013

[19] Root Kustritz MV (2014). Canine theriogenology for dog enthusiasts: teaching methodology and outcomes in a massive open online course (MOOC). J Vet Med Educ.

[20] Jordan K (2014). Initial trends in enrolment and completion of massive open online courses. Int Rev Res Open Dist Learn.

[21] Parr C. Not staying the course. Inside Higher Ed May 10, 2013. http://www.insidehighered.com/news/2013/05/10/new-study-low-mooc-completion-rate

[22] Melkun CH (2012). Nontraditional students online: composition, collaboration, and community. J Cont High Educ.

[23] Seaton DT, Bergner Y, Chuang I, Mitros P, Pritchard DE (2014). Who does what in a massive open online course?. Comm ACM.

[24] Toven-Lindsey B, Rhoads RA, Lozano JB (2015). Virtually unlimited classrooms: Pedagogical practices in massive open online courses. Internet High Educ.

[25] Pantò E, Comas-Quinn A (2013). The challenge of open education. J E- Learn Knowl Soc.

[26] Kyndt E, Dochy F, Onghena P, Baert H (2013). The learning intentions of low-qualified employees: a multilevel approach. Adult Educ Q.

[27] Cercone K (2008). Characteristics of adult learners with implications for online learning design. AACE J.

[28] Dixon R, Dixon K, Siragusa L. Individuals’ perceptions of online environments: what adult learners are telling us. In: ICT: providing choices for learners and learning. Proceedings ascilite Singapore 2007. http://www.ascilite.org.au/conferences/singapore07/procs/dixon.pdf

[29] Knowles MS (1980). The Modern Practice of Adult Education: From Pedagogy to Andragogy.

[30] Ross-Gordon JM (2011). Research on adult learners: supporting the needs of a student population that is no longer traditional. Peer Rev.

[31] Čermáková P, Fereshtehnejad S-M, Johnell K, Winblad B, Eriksdotter M, Religa D (2014). Cardiovascular medication burden in dementia disorders: a nationwide study of 19,743 dementia patients in the Swedish Dementia Registry. Alzheimer’s Res Ther.

[32] Waugh A, Austin A, Manthorpe J, Fox C, Stephens B, Robinson L (2013). Designing a complex intervention for dementia case management in primary care. BMC Fam Pract.

[33] World Health Organization (2012). Dementia: A Public Health Priority.

[34] Razavi S, Staav S (2010). Underpaid and overworked: A cross-national perspective on care workers. Int Labour Rev.

[35] Robinson A, Eccleston C, Annear M, Elliott K-E, Andrews S, Stirling C (2014). Who knows, who cares? Dementia knowledge among nurses, care workers, and family members of people living with dementia. J Palliat Care.

[36] King C, Robinson A, Vickers J (2014). Targeted MOOC captivates students. Nature.

[37] King C, Doherty K, Kelder J-A, McInerney F, Walls J, Robinson A (2014). “Fit for Purpose”: a cohort-centric approach to MOOC design. RUSC Univ Knowl Soc J.

[38] King C, Kelder J-A, Doherty K, Phillips R, McInerney F, Walls J (2014). Designing for quality: the Understanding Dementia MOOC. Electron J e Learn.

[39] MOOC Research. http://www.moocresearch.com/mooc-research-initiative-grantees

[40] Hollands FM, Tirthali D. MOOCs: Expectations and reality. Center for Benefit-Cost Studies of Education, Teachers College, Columbia University 2014. At: http://www.academicpartnerships.com/sites/default/files/MOOCs_Expectations_and_Reality.pdf

[41] Rodriguez O. Vast lurker and no-lurker participation in open online courses: MOOCs and the AI Stanford like courses respectively. http://cor-ar.blogspot.com.au/2012/03/moocs-and-ai-course-vast-lurker-and-no.html, March 2, 2012

